# Molecular Mechanisms Lead to Sex-Specific COVID-19 Prognosis and Targeted Therapies

**DOI:** 10.3389/fmed.2020.589060

**Published:** 2020-12-08

**Authors:** Thushara Galbadage, Brent M. Peterson, Jeffrey S. Wang, Avishka Jayasekara, Danny A. Ramirez, Joseph Awada, John P. Walsh, Richard S. Gunasekera

**Affiliations:** ^1^Department of Kinesiology and Health Science, Biola University, La Mirada, CA, United States; ^2^Department of Infectious Diseases, Southern California Permanente Medical Group, Pasadena, CA, United States; ^3^Leonard Davis School of Gerontology, University of Southern California, Los Angeles, CA, United States; ^4^Department of Chemistry, Physics, and Engineering, Biola University, La Mirada, CA, United States

**Keywords:** SARS-CoV-2, coronavirus, male, sex, ACE2, immunity, endocrine, androgen

## Abstract

Clinical and epidemiological studies have identified male sex as an important risk factor for COVID-19 clinical outcomes and mortality. This raises the question as to how this risk factor can be addressed in the prognosis, clinical management, and the treatment of patients with Coronavirus disease 2019 (COVID-19). Currently, there are no guidelines or protocols to help alter the course of sex-specific COVID-19 prognosis, especially in severe disease presentations. This is partly due to the lack of research studies characterizing the differences in male vs. female host response to the severe acute respiratory syndrome Coronavirus-2 (SARS-CoV-2) infection and a lack of a well-rounded understanding of the molecular mechanisms involved. Here, we discuss three distinct but interconnected molecular-level differences in males and females that likely play an essential role in the COVID-19 prognosis. We review interactions of SARS-CoV-2 with host cell angiotensin-converting enzyme 2 (ACE2) in the viral entry between males vs. females and discuss the differential regulation of the renin-angiotensin system (RAS) between the two sexes. Next, we present immune response disparities and how immune function and endocrine regulation may render males increasingly vulnerable to severe COVID-19. We describe the interconnected roles of these three regulatory systems in males and females in response to SARS-CoV-2 infection. Finally, we highlight the clinical implications of these mechanisms to patients with COVID-19 and propose putative targeted therapies that can help reduce COVID-19 severity in those critically ill.

## Introduction

The severe acute respiratory syndrome Coronavirus 2 (SARS-CoV-2) infections elicit a wide variety of clinical outcomes among patients. As the Coronavirus disease 2019 (COVID-19) pandemic unfolds, a greater understanding of disease-related risk factors is developing. For example, primary risk factors for severe COVID-19 are advanced age, comorbidities including chronic respiratory disease, cardiovascular disease, diabetes, and hypertension (1–4). Additionally, multiple studies stratifying COVID-19 cases and mortality rates by sex have demonstrated sex-related divergence between males and females in severe and fatal cases (2, 5–9). More recently, systematic reviews of COVID-19 clinical studies have helped establish the male sex as a substantial risk factor for poor prognostic outcomes of COVID-19 and higher mortality rates (10–12).

Studies have also noted that social risk factors such as smoking and tobacco use may predispose males to more severe forms of COVID-19 (13–16). We examined sex-specific COVID-19 clinical outcomes and observed that the male sex was a distinct risk factor across the world, irrespective of region or country (10). While social factors may play a role in Coronavirus disease severity, mounting evidence makes it undeniable that there are likely sex-specific molecular mechanistic differences that make males more susceptible to severe COVID-19. There are inherent sex-specific differences in males vs. females. These differences may provide a more comprehensive explanation for observed sex-specific COVID-19 clinical outcomes. For example, at the molecular level, males and females respond to various infections differently in terms of immune response and regulation of endocrine function (17–21). Currently, however, sex-specific host response differences to SARS-CoV-2 at the molecular level have yet to be clearly defined.

**Figure 1:**
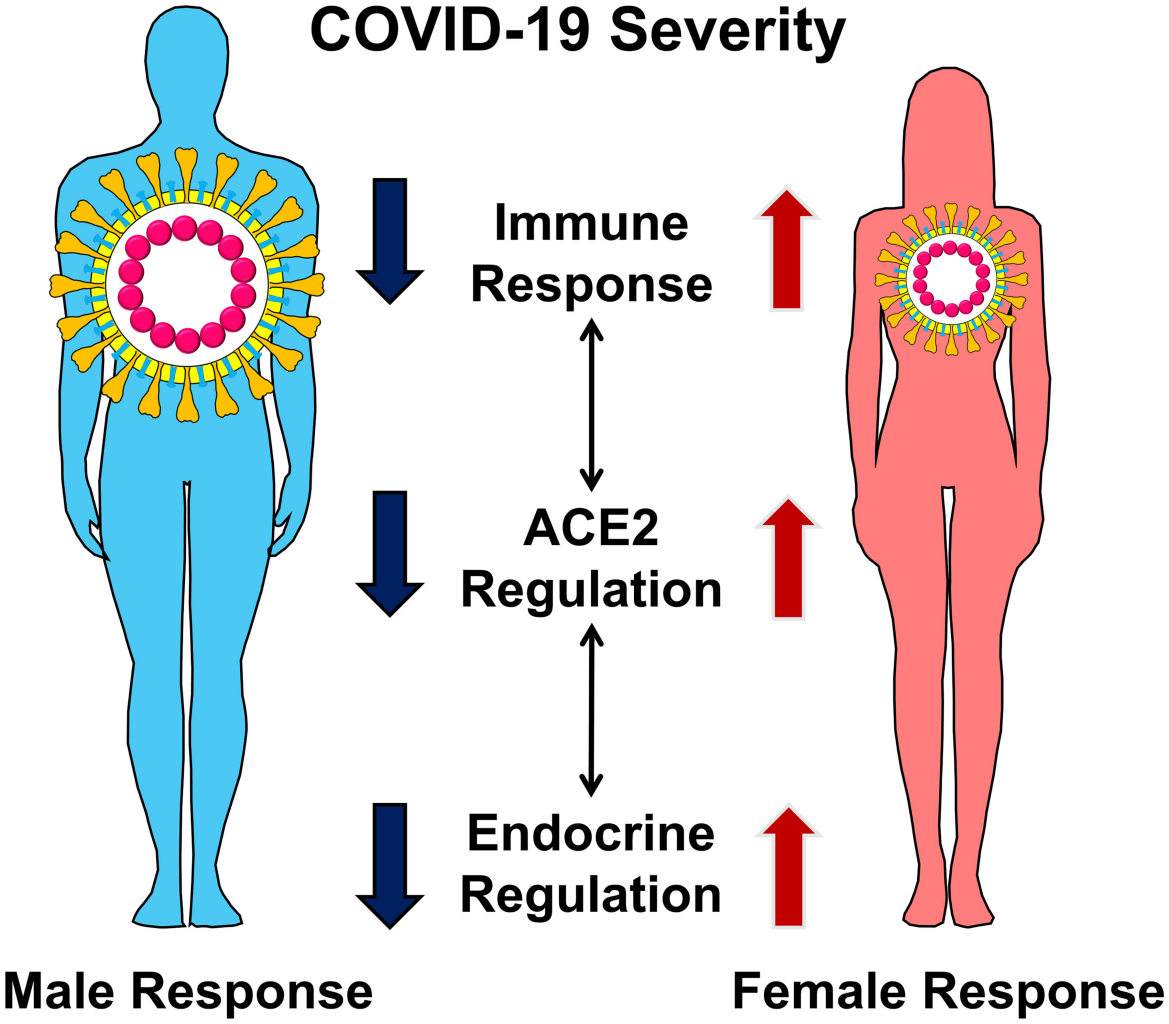
Male and female display differences in COVID-19 severity. The differences in disease severity can be explained by sex-specific differences in the regulation of ACE2, immune response, and endocrine regulation. These distinct but interconnected molecular mechanisms lead to severe COVID-19 in men.

Our review examines three categories of molecular-level differences in males and females that are likely to lead to severe COVID-19 disease in males. These include (1) the molecular interaction of angiotensin-converting enzyme 2 (ACE2) with SARS-CoV-2 and its modulation within the renin-angiotensin system (RAS), (2) sex-related endocrine differences in immune responses to pathogens, and (3) the effects of estrogen and androgens on regulation of ACE2 and RAS (Figure 1). Importantly, a better understanding of these particularly unique sex-specific differences in the severity of COVID-19 could result in the use of targeted therapy by clinicians to help avert life-threatening diseases.

## ACE2 Acts as the Functional Receptors for Viral Cell Entry

ACE2 is a homolog of the angiotensin-converting enzyme (ACE) that acts to modulate RAS by cleaving both angiotensin I (Ang-I) and angiotensin II (Ang-II) (22, 23). With the COVID-19 pandemic, the role of ACE2 in the pathogenesis of SARS-CoV-2 has gained broad interest, especially for its connection with the RAS and its clinical implications (24). SARS-CoV-2 shares high amino acid sequence homology with SARS-CoV-1 from the 2002–2003 SARS outbreak. SARS-CoV-1 enters into host cells using the viral spike (S) protein as a ligand to the host ACE2 receptor (25–27). Similarly, ACE2 is the functional host receptor for SARS-CoV-2, acting as an epithelial entry point in the respiratory tract (28). The SARS-CoV-2 S protein engages the ACE2 and uses the host serine protease TMPRSS2 for S-priming, fusion, and entry. Binding of SARS-CoV-2 to ACE2 receptors reportedly occurs with 10–20 times higher binding affinity than SARS-CoV-1, making it more virulent and transmissible (28–30). Thus, increased ACE2 expression may facilitate the viral cell entry process. The internalization of ACE2 with SARS-CoV-2 leads to a decrease in the amount of ACE2 receptors on the cell surface, and this affects the delicate RAS balance (31). ACE2 and RAS are essential for blood pressure regulation and electrolyte homeostasis (32). This connection led clinicians to be concerned about the use of RAS system modulating medications like angiotensin receptor blockers (ARBs) and ACE inhibitors as they managed their patients (33, 34). The safe use of these hypertensive mediations amidst the COVID-19 pandemic is currently an area of active research (35).

## Males and Females Differentially Express ACE2 Leading to Different COVID-19 Outcomes

Androgens play a role in the RAS by promoting the expression of angiotensinogen and increases the plasma activity of renin (36). In contrast, estrogen downregulates the expression of AT1R, ACE, and decreases the plasma activity of renin (37). Besides modulating ACE activity, estrogen upregulates the expression of ACE2, AT2R, and the mitochondrial assembly receptor (Mas R) (19). This is a critical difference in the regulation of the RAS by androgen and estrogen. The activation of the ACE2 and AT1R axis by androgen leads to inflammation, hypertension, vasoconstriction, fibrosis, and cell proliferation (38, 39). In comparison, the activation of the alternative angiotensin (1-7) [Ang-(1-7)], AT2R, and Mas R axis by estrogen leads to protective effects including anti-inflammation, hypotension, vasodilatation, and apoptosis (40, 41). Ang-II levels among patients with COVID-19 is strongly associated with viral load and lung injury. Plasma samples of Ang-II in patients with COVID-19 revealed substantial elevations and was associated with viral load and lung injury (42). Clinically elevated Ang-II can result in detrimental physiological effects, stimulating the adrenal gland and, subsequently, increasing blood pressure. SARS-CoV-2 uses ACE2 receptors for viral cell entry and thereby downregulates cell surface ACE2 (28). Downregulating ACE2 reduces the conversion of Ang-II to Ang-(1-7), which can lead to the increased activity of AT1R its downstream effects.

Regulation of RAS has been demonstrated to differ between males and females in animal models. For example, in a study, male rats expressed higher Ang-II and angiotensin II receptor type 1 (AT1R) as opposed to female rats with higher Angiotensin II Receptor Type 2 (AT2R) and Ang-(1-7) (43). Additionally, orchiectomy decreased enzymatic function in male mice, while ovariectomy increased hypertrophy and ACE2 in female mice. Young-adult rats (3 months old) had a higher ACE2 expression compared to older rats (24 months old), respectively (44). These observations may help explain the paradoxical decrease in the level of ACE2 receptors observed in lung tissue of patients infected with SARS-CoV-2. Interestingly, observations of reduced ACE2 expression in patients who were infected with SARS-CoV-1 may suggest mechanisms that lead to SARS (45–47). These results further support that estrogen and androgens play an essential role in the regulation of the RAS.

Males express more ACE and ACE2 and thus, SARS-CoV-2 binding to ACE2 and its subsequent internalization removing this critical blood pressure reducing mechanism (48). The balance of ACE/ACE2 is toward ACE2 in females, and this relative difference in expression may convey protection by reducing blood pressure (49). These sex-differences in ACE2 are likely contributing to sex differences observed in COVID-19-related infection, morbidity, and mortality (48, 49). Female-specific immune response and ACE2 regulation also work together to maintain both immune function and blood pressure homeostasis (50).

## Sex Differences in Immune Response Lead to Differences in Disease Prognosis

Men are more susceptible to viral, bacterial, fungal, and parasitic infections, while women are more likely to suffer from autoimmune disease (18, 51–54). Women also mount superior immunity in response to vaccinations (52, 55, 56). Sex-based vulnerability to SARS-Cov-2 infection and the severity of COVID-19 disease are likely linked to females having stronger innate and adaptive immunity and associated sex-differences in cytokine release and inflammatory response (48). Sex-based differences in cellular immune responses and inflammation are linked to X chromosome encoded genes, and later on, the differential impact of estrogen vs. androgen on immune function (18, 54, 56). Several genes found on both X and Y chromosomes and sex hormones direct hematopoietic myeloid and lymphoid lineage profiles, subsequent cell development and surface protein expression, and cytokine production (18, 54, 56). Females show higher CD4+ cell counts and a higher CD4+/CD8+ ratio, with more active and more numerous cytotoxic T- and T-suppressor cells (18). For SARS-CoV-2, a more inhibited adaptive immune response in females who have more T-cells is represented by lower mortality rates of females in comparison with males (6, 8, 9).

Several genes influencing immune cell function, and immune cell protein expression are located on X chromosomes (51, 54). These X-linked genes control the expression of pattern recognition receptors like TLR-7 and TLR-8, CD132, and CD40 in both innate and adaptive immune cells and modulators of NF-kappa-B transcription factor (51, 54). The X chromosome also contains a large number of micro-RNAs (miRNAs) involved in the modulation of immune function and the gene for the androgen receptor (AR) (Xq12 location) (54). Besides the male-specific functions of the AR, it is vital in the modulation of antibody production and the transcription of the serine protease TMPRSS2 (57). Therefore, AR plays a critical role in the SARS-CoV-2 viral cell entry, as TMPRSS2 is required for the priming of the S protein before cell entry (28). Males having a higher level of AR compared to females may result in an increased level of COVID-19 susceptibility.

## Endocrine Regulation of the Immune Response in Males and Females

In general, estrogen acts to activate the immune system, while androgens act to inhibit immune function (54). Estrogen receptors (ERα and ERβ) are found on hematopoietic progenitor cells, and they show unique expression in differentiated lymphocytes (T- and B-cells) and myeloid cells (mast cells, macrophages, dendritic cells, and natural killer cells) (56, 58). Estrogen binding to receptors in tissue invaded by the immune system can also reduce oxidative and apoptotic damage that can serve as a trigger for initiating platelet aggregation and the coagulation cascade (53). Females show higher myeloid phagocytic activity, and myeloid cell antigen presentation is superior to that found in males (18). Estrogen modulates the function of both CD4+ and CD8+ T-cells and myeloid cell lines. Estrogen increases the expression and release of TH1 pro-inflammatory cytokines (IL-12, TNF-α, IFN-γ) and reduces the release of TH2 anti-inflammatory cytokines IL-10, IL-4 and TGF-β in a concentration-dependent manner (54, 58). Estrogen and androgen bind to innate immune cells resulting in activating and suppressing effects, respectively (53, 54). Analysis of innate immunity shows female dendritic cells are more responsive to viral infections, and they show increased production of INF-α (51, 54). This female-linked difference in dendritic cell function is influenced by estrogen modulation of antigen recognizing Toll-like receptor 7 (TLR7). Many of these sex-based differences are retained after menopause and seen before puberty, indicating a pivotal role played by X chromosome gene expression independent of hormone receptor activation [54). This regulation highlights the interconnected role of the immune response and the endocrine regulation that leads to sex-specific disease outcomes (43, 52, 59).

## Sex-Specific Molecular Mechanisms Regulate COVID-19 Severity

**Figure 2:**
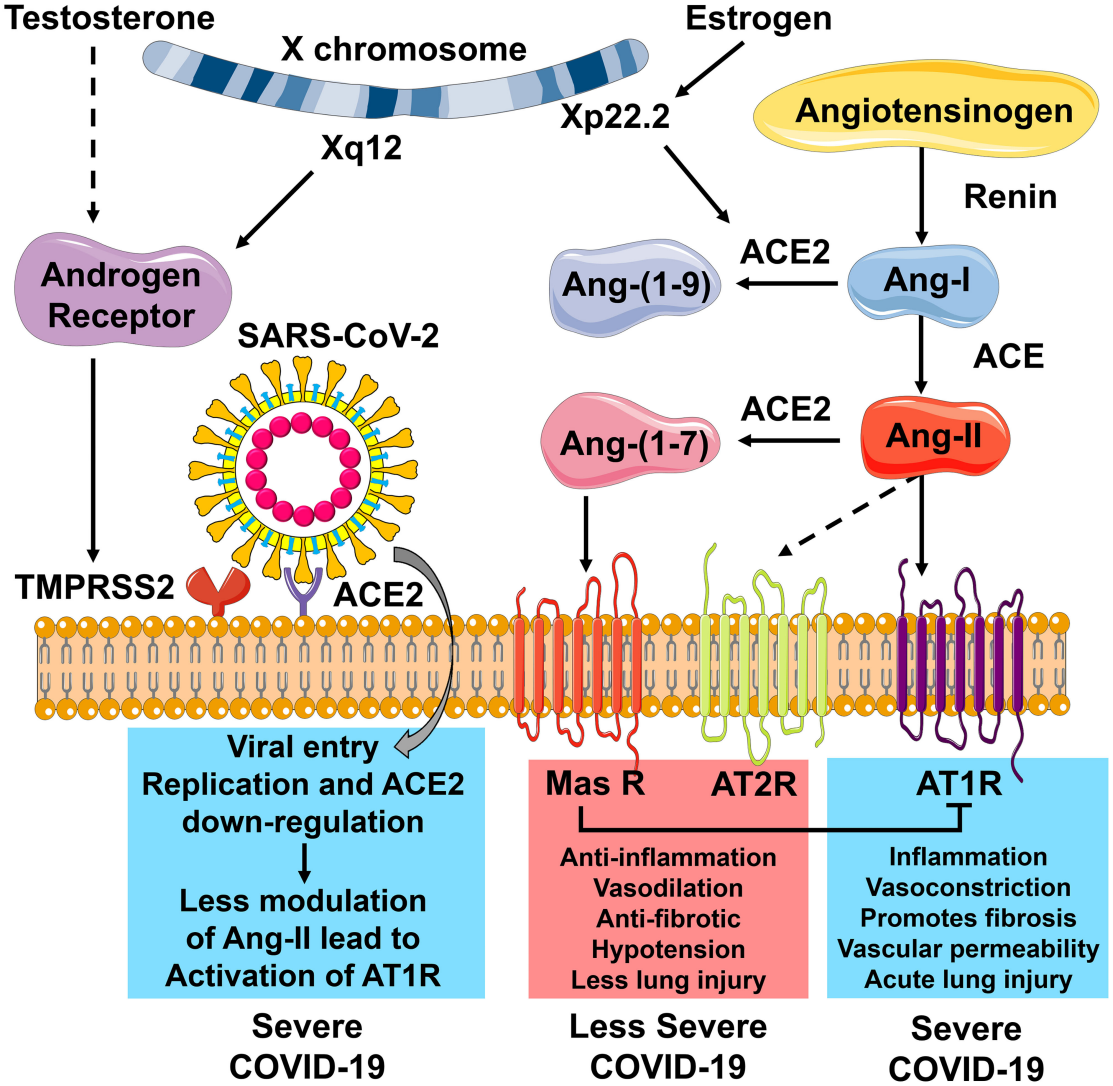
The role of ACE2 receptors in modulating COVID-19 severity. Angiotensin-converting enzyme 2 (ACE2) plays a central role in the observed sex-specific disparities in COVID-19 severity. ACE2 is the receptor for viral cell entry. Transmembrane protease, serine 2 (TMPRSS2), is needed for priming of the viral S protein to allow viral cell entry. The transcription of TMPRSS2 is under the control of the Androgen Receptor (AR), which is located on the X chromosome (Xq12). With SARS-CoV-2 entry into the host cell, ACE2 is downregulated. In the absence of ACE2, angiotensin-II (Ang-II) cannot be cleaved to give angiotensin-(1-7) [Ang-(1-7)], and the action of Ang-II increases. Activation of angiotensin type 1 receptor (AT1R) by Ang-II leads to more inflammation, vasoconstriction, fibrosis, vascular permeability, and acute lung injury and results in severe COVID-19. The ACE2 gene is located on the X chromosome (Xp22.2) and is regulated by estrogen. Estrogen causes increased levels of ACE2, which cleaves Ang-II to Ang-(1-7). Ang-(1-7) activates the mitochondrial assembly receptor (Mas R). This causes anti-inflammation, vasodilation, reduced fibrosis, hypotension, and less lung injury, leading to a less severe form of COVID-19. Outcomes highlighted in blue are associated more with males, and those highlighted in red are associated more with females. This figure was created with the images available at Servier Medical Art, licensed under a Creative Commons Attribution 3.0 Unported License.

The RAS function, immune response, and the endocrine regulation are interconnected in their response to SARS-CoV-2 (Figure 2). ACE2 plays a central role in the observed sex-specific disparities in COVID-19 severity. ACE2 is the receptor for viral cell entry, and TMPRSS2 is essential for priming of the viral S protein before viral cell entry can happen. The transcription of TMPRSS2 is under the control of the AR, which is located on the X chromosome (Xq12). With the entry of SARS-CoV-2, ACE2 is downregulated, and the action of Ang-II increases. This leads to greater inflammation, fibrosis, and acute lung injury, causing severe COVID-19 (Figure 2). The ACE2 gene is also located on the X chromosome (Xp22.2) and is regulated by estrogen. Estrogen causes increased levels of ACE2, which cleaves Ang-II to Ang-(1-7). ACE2 causes the activation of the Ang-(1-7)/Mas R/AT2R axis and the inhibition of the ACE/Ang-II/AT1R axis. Therefore, increased levels of ACE2 cause an anti-inflammatory response, reduce fibrosis and protects the lungs from acute injury. This leads to a less severe form of COVID-19 (Figure 2). These sex-specific molecular mechanisms involved in SARS-CoV-2 infections offer potential targets for therapy to help reduce the severity of the disease.

## Clinical Perspective on COVID-19 Prognosis and Current Therapy

While any person can acquire COVID-19, there tends to be a higher rate of disease in males compared to females, and this holds regardless of where on the spectrum of clinical severity the disease (6, 8, 10). The predominantly described symptoms of COVID-19 usually involve some combination of fever, cough, and shortness of breath. However, other symptoms have also been described, including headache, chills, fatigue, anosmia, sore throat, congestion, rhinorrhea, nausea, diarrhea, and myalgias (60). In the majority of patients, COVID-19 symptoms stay mild enough to allow for recovery at home. Still, a significant minority of patients will progress in their symptoms to the point where they require hospitalization. Patients who suffer from the severe form of the disease may have a hyper-inflammatory response to the viral infection, also described as a “cytokine storm,” which then leads to some of the more catastrophic clinical findings, including acute respiratory distress syndrome ARDS (61).

A variety of pharmacotherapies have been tried that target both the virus itself and the immune response. Some of the most promising include remdesivir and dexamethasone. Remdesivir is an anti-viral developed initially in 2015 for the treatment of Ebola. Remdesivir is a prodrug whose active metabolites interfere with the action of viral RNA-dependent RNA polymerase, ultimately causing a decrease in viral RNA production. Preliminary results from the adaptive COVID-19 clinical trial sponsored by the National Institute of Allergy and Infectious Diseases demonstrate a significant reduction in time to recovery for patients on remdesivir when compared to placebo and a trend toward improved mortality (62, 63). Dexamethasone, a synthetic corticosteroid with potential utility in tamping down inflammation, was found in the RECOVERY trial was found to have reduced deaths by one-third in ventilated patients and by one fifth in other patients receiving oxygen only (64). Patients who do poorly, as described above, tend to be male and tend to have underlying comorbidities. Longer than average days of mechanical ventilation have been described in these severely ill patients. Death is often from progressive, refractory pulmonary failure, but other organ systems have also been seen to be affected, including renal failure, cardiac injury, and thromboembolic events. Clinicians will benefit knowing male patients presenting with COVID-19 have a higher chance of experiencing rapid clinical deterioration compared to their female counterparts.

## Anti-Androgens as Potential Targeted Therapy for COVID-19

With increasing evidence of sex-specific COVID-19 prognosis, there is a renewed interest in looking into the use of targeted clinical therapy. Anti-androgen therapy, including the use of 5-alpha reductase inhibitors in male patients, is currently being studied (65, 66). In a study looking at prostate cancer patients' risk of contracting COVID-19, those that were on androgen-deprivation therapies (ADTs) showed a partial protective effect (67). Since AR controls the transcription of TMPRSS2, this may serve as a potential targeted therapy to reduce the severity of COVID-19 in male patients. This ultimately begs the question of whether anti-androgen therapies should be given to male patients with COVID-19.

Several dietary compounds have been proven to work as 5-alpha reductase inhibitors and anti-androgenic therapeutics. Curcumin, the active ingredient in turmeric; lycopene, the red pigment found in tomato; and capsaicin, the chemical found in chili peppers, are known androgen inhibitors via their inhibitory properties in the metabolism of dihydroxy testosterone (68–72). These are well known also due to their preventative abilities to prevent prostate disease. Therefore, given the involvement of AR in SARS-CoV-2 pathogenesis, these compounds may have some preventative potential against COVID-19. Our review puts the current research literature in perspective to help emphasize the importance of sex-specific differences in the clinical management of COVID-19 patients. While it may be too early to determine the effectiveness of such treatment, it highlights the evolution of medical practice in the treatment and prevention of infectious diseases to include sex-specific differences in pathogenesis.

## Author Contributions

All authors listed have made a substantial, direct and intellectual contribution to the work, and approved it for publication.

## Conflict of Interest

JW was employed by company Southern California Permanente Medical Group.

The remaining authors declare that the research was conducted in the absence of any commercial or financial relationships that could be construed as a potential conflict of interest.
